# CSF pTau181 predicts asymmetric hippocampal atrophy in cognitively unimpaired older adults

**DOI:** 10.1016/j.tjpad.2026.100690

**Published:** 2026-11-04

**Authors:** Elham Ghanbarian, Lukai Zheng, Tianchen Qian, Crystal M. Glover, S. Ahmad Sajjadi, Maria M. Corrada, Joshua D. Grill, Ali Ezzati

**Affiliations:** a Department of Neurology, University of California, Irvine, CA, USA; b Center for Design, Discovery, and Data Analytics (3D Center) in Neurological Disorders, University of California, Irvine, CA, USA; c Department of Statistics, University of California, Irvine, CA, USA; d Alzheimer's Disease Research Center, Institute for Memory Impairments and Neurological Disorders (UCI MIND), University of California, Irvine, CA, USA; e Department of Psychiatry and Human Behavior, University of California, Irvine, CA, USA; f Department of Epidemiology and Biostatistics, University of California, Irvine, CA, USA

**Keywords:** Alzheimer’s disease, Hippocampal asymmetry, Hippocampal atrophy, Phosphorylated tau 181 (pTau181), Amyloid-β 42 (Aβ42)

## Abstract

**Background:**

Hippocampal atrophy is a core marker of neurodegeneration in dementia, particularly in Alzheimer’s disease (AD). However, most studies focus on total hippocampal volume loss and overlook hemispheric asymmetry, which may reflect distinct biological processes. While tau pathology is closely linked to medial temporal lobe degeneration, it remains unclear whether tau is associated with asymmetric patterns of hippocampal atrophy.

**Methods:**

We analyzed 483 cognitively unimpaired participants from the Alzheimer’s Disease Neuroimaging Initiative (ADNI) with baseline cerebrospinal fluid (CSF) phosphorylated tau (pTau181) and amyloid-β (Aβ42) measurements and longitudinal structural MRI data over 10 years of follow-up. Total hippocampal volume and hemispheric asymmetry, defined as the absolute difference between left and right hippocampal volumes (|L−R|), were quantified at each visit. Linear mixed-effects models assessed associations between baseline CSF biomarkers and longitudinal changes in hippocampal asymmetry, adjusting for demographic factors, APOE ε4 status, and baseline hippocampal volume.

**Results:**

Higher baseline CSF pTau181 was associated with greater increases in hippocampal asymmetry over time (β = 1.20, SE = 0.43, *p* = 0.006). This association remained significant after additional adjustment for total hippocampal volume and baseline CSF Aβ42. CSF total tau was highly correlated with pTau181 (Spearman’s ρ =0.98, *p* < 0.001) and showed comparable associations with hippocampal measures. In contrast, baseline Aβ42 was not associated with subsequent changes in hippocampal asymmetry. Both pTau181 and Aβ42 were associated with faster decline in total hippocampal volume. In amyloid-stratified analyses, pTau181 was associated with increasing hippocampal asymmetry only among amyloid-negative individuals, whereas its association with total hippocampal atrophy was observed primarily in amyloid-positive individuals.

**Conclusions:**

CSF pTau181 is associated not only with overall hippocampal atrophy but also with progressive hippocampal asymmetry, suggesting that tau-related neurodegeneration may manifest as both magnitude and imbalance of tissue loss. These findings support hippocampal asymmetry as a complementary neuroimaging marker that may capture medial temporal lobe neurodegeneration in cognitively unimpaired older adults, with potential applications in early disease risk stratification, longitudinal disease monitoring, and the identification of biologically distinct participants for future clinical trials.

## 1 Introduction

Hippocampal atrophy is one of the most robust and widely used neuroimaging markers of Alzheimer’s disease (AD), reflecting neurodegeneration across both preclinical and clinical stages [1]. Progressive volume loss within medial temporal lobe (MTL) structures, particularly the hippocampus and entorhinal cortex, closely tracks cognitive decline and risk of progression to dementia [2] MRI-derived measures of hippocampal atrophy have been consistently shown to correlate with underlying neuropathology, supporting its role as valid in-vivo biomarkers of disease progression [3].

While overall hippocampal atrophy is well established as a diagnostic and prognostic marker of AD, less is known about hippocampal hemispheric asymmetry. Most prior work has treated total hippocampal volume as the primary structural summary of disease-related degeneration. However, as a bilateral summary measure, total volume may obscure an important feature of MTL neurodegeneration: its lateralization. If degeneration does not occur symmetrically across the two hippocampi, then the overall magnitude of volume loss may fail to capture asymmetric atrophy, which may provide distinct and biologically meaningful information [4].

Limited prior work on hippocampal asymmetry in AD has been mixed, suggesting that asymmetry is not simply a universal feature of AD severity but may instead reflect specific biological subtypes or pathological heterogeneity [[5], [6], [7], [8], [9]] Recent work from our group has shown that while total hippocampal volume is associated with cognitive performance in multiple domains, greater left-right hippocampal asymmetry is primarily associated with accelerated memory decline, especially among amyloid-negative individuals [4]. These findings suggest that hippocampal asymmetry may provide complementary information beyond total hippocampal volume and capture non-amyloid neurodegenerative pathways. Furthermore, conditions not primarily defined by amyloid pathology, such as hippocampal sclerosis of aging (HSA), often associated with limbic-predominant age-related TDP-43 encephalopathy (LATE) neuropathological changes, can produce marked asymmetric hippocampal atrophy [10,11]

Accumulating evidence suggests that tau pathology, rather than amyloid burden, is more directly linked to neurodegeneration and atrophy in AD [12,13] Tau deposition begins within MTL structures, and regional atrophy closely mirrors the pattern of tau spread in these regions, whereas amyloid accumulation does not show the same tight coupling with atrophy [12,14] In preclinical AD, accelerated longitudinal atrophy of MTL, including hippocampus, has been reported in individuals with elevated tau, supporting that hippocampal atrophy emerges in the context of tau pathology [13,15] In particular, post-mortem studies have demonstrated that phosphorylated tau deposition is strongly associated with hippocampal atrophy [16,17]

Although the role of tau pathology in MTL, particularly hippocampal, atrophy is well established, it remains unclear whether tau is associated primarily with overall hippocampal volume loss or also with progressive hippocampal asymmetry. If tau contributes to asymmetric neurodegeneration independent of total hippocampal volume, this suggests that tau pathology influences not only the magnitude of tissue loss but also its spatial distribution. Such findings could improve our understanding of the biological heterogeneity of AD, facilitate the identification of individuals at increased risk of progressive neurodegeneration, and support more biologically informed participant selection in clinical trials.

While the relationship between AD biomarkers and overall hippocampal atrophy has been extensively studied, their association with longitudinal changes in hippocampal asymmetry has not been previously investigated. Because there was insufficient evidence to propose a specific directional hypothesis, we therefore conducted an exploratory investigation. In this study, we examined whether baseline CSF AD biomarkers, Aβ42 and pTau181, were associated with longitudinal changes in both total hippocampal volume and hippocampal asymmetry in cognitively unimpaired older adults. We focused on cognitively unimpaired individuals to determine whether these biomarkers are associated with subtle structural changes that precede substantial neurodegeneration and cognitive impairment.

## 2 Methods

### 2.1 Participants

#### 2.1.1 ADNI study design

Data used in the preparation of this article were obtained from the Alzheimer’s Disease Neuroimaging Initiative (ADNI) database (adni.loni.usc.edu). The ADNI was launched in 2003 as a public-private partnership, led by Principal Investigator Michael W. Weiner, MD. The primary goal of ADNI has been to test whether serial magnetic resonance imaging (MRI), positron emission tomography (PET), other biological markers, and clinical and neuropsychological assessment can be combined to measure the progression of mild cognitive impairment (MCI) and early AD. Importantly, ADNI diagnoses are based on standardized clinical criteria and do not require pathological confirmation, allowing for etiologic heterogeneity at the clinical endpoint. ADNI data collection was approved by the institutional review boards of all participating institutions and informed written consent was obtained from all participants. Detailed information on measures and methods of assessment in the ADNI project is available at http://www.adni.loni.usc.edu.

#### 2.1.2 Study participants

We included participants from ADNI (ADNI 1, ADNI GO, ADNI 2, and ADNI 3) who met the following criteria at baseline: (1) cognitively unimpaired (CU) status, defined as an MMSE score of 24 or higher, a CDR score of 0, and no evidence of depression; (2) availability of at least two longitudinal T1-weighted MRI scans processed through the ADNI FreeSurfer pipeline; and (3) availability of baseline CSF concentrations of amyloid-β (Aβ42) and phosphorylated tau at threonine 181 (pTau181). Participants with a baseline diagnosis of mild cognitive impairment or dementia were excluded. After applying these criteria, 483 CU participants were included in the study.

Availability of tau PET data was not part of the participant inclusion criteria. However, baseline tau PET data were available for a subset of 130 participants and were used in a secondary analysis to examine the association between CSF pTau181 and cerebral tau deposition.

### 2.2 Study measures and outcomes

#### 2.2.1 MRI and hippocampal asymmetry

Structural MRI data were preprocessed using FreeSurfer (http://surfer.nmr.mgh.harvard.edu/) by the ADNI MRI Core as part of the ADNI standard pipelinesset. FreeSurfer methods for identifying and calculation of regional brain volumes have been described in detail previously [18]

Left and right hippocampal volumes were adjusted for intracranial volume (ICV) by dividing each hippocampal volume by the participant’s ICV and multiplying the resulting ratio by the mean ICV of the study population. This rescaling yields ICV-adjusted hippocampal volumes expressed in cm³, facilitating interpretation while accounting for individual differences in head size. Total hippocampal volume was defined as the sum of the adjusted left and right hippocampal volumes (L + R), and hippocampal asymmetry as the absolute difference between the adjusted left and right hippocampal volumes (|L − R|). The absolute difference was used to quantify the magnitude of hemispheric imbalance, as our objective was to examine whether greater asymmetry itself, rather than its direction, was associated with CSF biomarkers.

#### 2.2.2 CSF biomarkers

CSF samples were batch processed by the ADNI Biomarker Core at the University of Pennsylvania School of Medicine using Roche Elecsys assay [19] Baseline CSF pTau181 and Aβ42 levels were included as continuous variables in all models. For descriptive purposes and stratified models, amyloid positivity was defined as CSF Aβ42 < 977 pg/mL, and pTau181 positivity was defined as CSF pTau181 > 27 pg/mL, consistent with validated ADNI cutpoints [20]. Additionally, baseline total tau (t-tau) was analyzed as a continuous variable for comparison with pTau181.

#### 2.2.3 Tau PET imaging

To provide biological context for the primary analyses and determine the extent to which CSF pTau181 reflected in vivo cerebral tau pathology within this cohort, we evaluated the association between CSF p-tau181 and tau PET measures in the subset of participants with available tau PET data. Tau PET ([18F]flortaucipir, AV-1451) data were obtained from the ADNI database in fully preprocessed form using the standardized ADNI pipeline. T1-weighted MRI scans were processed with FreeSurfer (v5.3) to define regions of interest (ROIs), and PET images were co-registered to the corresponding MRI using SPM5. SUVR values were calculated by normalizing regional tracer uptake to inferior cerebellar gray matter. A temporal meta-ROI was defined as the volume-weighted average of bilateral entorhinal, amygdala, fusiform, inferior temporal, and middle temporal regions. Tau positivity was determined using a previously validated SUVR threshold of ≥1.37 for the meta-ROI [21]

#### 2.2.4 Statistical analysis

Differences in participant characteristics stratified by pTau181 and Aβ42 status were tested using independent-sample t tests for continuous variables and chi-squared test for categorical variables. Associations between CSF pTau181 and t-tau, as well as between these biomarkers and tau PET SUVR were assessed using Spearman rank correlation. Additionally, partial correlation adjusted for age, sex and amyloid burden were used to evaluate the associations between CSF tau biomarkers and tau PET. Dichotomized variables for pTau181 and Aβ42 were defined based on the cutoffs described in section 2.2.2. APOE ε4 status was coded as a binary variable (carrier vs. non-carrier), with carriers defined as participants with at least one ε4 allele.

To evaluate the associations between baseline pTau181 and Aβ42 and longitudinal changes in total hippocampal volume and asymmetry, we used linear mixed-effects (LME) models. Separate models were fit for hippocampal asymmetry and total volume. For hippocampal asymmetry, Model 1 was adjusted for age, sex, education, APOE ε4 status, time, the baseline CSF biomarker (pTau181 or Aβ42), and the corresponding biomarker × time interaction. Model 2 additionally adjusted for baseline total hippocampal volume. Model 3 additionally adjusted for the other CSF biomarker (Aβ42 in pTau181 models and pTau181 in Aβ42 models) and its interaction with time. Similarly, for total hippocampal volume, we used 3 models. Model 1 was adjusted for age, sex, education, APOE ε4 status, time, the baseline CSF biomarker (pTau181 or Aβ42), and the corresponding biomarker × time interaction. Model 2 additionally adjusted for the alternate CSF biomarker and its interaction with time. Model 3 additionally adjusted for baseline hippocampal asymmetry.

Time was modeled as a continuous variable representing years since baseline visit. Random intercepts and slopes for time were included at the participant level to capture individual variability in baseline hippocampal asymmetry and its rate of change. The estimates for biomarker reflect the associations with baseline hippocampal asymmetry or baseline total volume (time 0), whereas the biomarker × time interactions reflect the associations with the rate of change in asymmetry over time or total volume over time.

Additionally, amyloid-stratified analyses were conducted to examine potential effect modification by baseline binary amyloid status. Separate LME models were fit within amyloid-positive and amyloid-negative groups using identical model specifications. Also, to evaluate the associations between pTau181 and longitudinal trajectories of left and right hippocampal volumes, we fitted separate LME models with left and right hippocampal volume as the respective outcomes. All statistical analyses were conducted in SPSS version 27 (IBM Corp, Armonk, NY, USA).

## 3 Results

### 3.1 Cohort characteristics

The study cohort consisted of 483 cognitively unimpaired individuals, of whom 275 (56.9%) were females, and the mean age was 72.9 ± 6.15 years (Table 1). The average total hippocampal volume in the entire sample was 7.62 ± 0.87 cm^3^, and the average hippocampal asymmetry was 0.22 ± 0.17 cm^3^. On average, the right hippocampus was larger than the left in the entire cohort (3.87 ± 0.47 vs 3.75 ± 0.44 cm^3^, paired t-test, *p* < 0.001).

**Table 1:** Baseline characteristics of all participants, overall and stratified by CSF pTau181 and Aβ42 status.

Characteristic	All	pTau181-	pTau181+	p value	Aβ42-	Aβ42+	p value
Participants, n (%)	483	378 (78.3)	105 (21.7)	-	314 (65.01)	169 (34.99)	-
Female Sex, n (%)	275 (56.9)	214 (56.6)	61 (58.1)	0.782	179 (57)	96 (56.8)	0.961
APOE ε4 carrier, n (%)	151 (31.3)	107 (28.3)	44 (41.9)	0.008	61 (19.4)	90 (53.3)	<0.001
Age, yr	72.90 (6.15)	72.19 (5.92)	75.44 (6.30)	<0.001	72.52 (6.12)	73.60 (6.15)	0.065
Education, yr	16.53 (2.52)	16.47 (2.53)	16.75 (2.47)	0.307	16.55 (2.54)	16.50 (2.48)	0.833
CSF Aβ42, pg/mL	1344.32 (664.97)	1336.06 (586.10)	1374.07 (896.09)	0.682	1690.82 (565.32)	700.54 (178.61)	<0.001
CSF pTau181, pg/mL	21.46 (8.96)	17.64 (4.49)	35.23 (7.36)	<0.001	20.55 (7.63)	23.16 (10.82)	0.006
CSF pTau181/Aβ42	0.021 (0.02)	0.016 (0.01)	0.038 (0.02)	<0.001	0.012 (0.01)	0.036 (0.02)	<0.001
CSF total tau, pg/mL	235.75 (86.1)	199.56 (48.13)	366.02 (63.80)	<0.001	233.59 (80.36)	239.75 (95.98)	0.478
Tau PET SUVR, temporal meta-ROI*	1.21 (0.15)	1.18 (0.12)	1.29 (0.21)	<0.001	1.18 (0.07)	1.26 (0.22)	0.026
Total Hippocampal Volume, cm^3^	7.62 (0.87)	7.66 (0.88)	7.46 (0.84)	0.041	7.64 (0.85)	7.59 (0.91)	0.561
Hippocampal Asymmetry, cm^3^	0.22 (0.17)	0.22 (0.16)	0.24 (0.18)	0.245	0.22 (0.17)	0.22 (0.17)	0.894

Continuous variables are expressed as mean (Standard deviation, SD), and categorical variables as counts (percentages). Amyloid positivity was defined as CSF Aβ42 < 977 pg/mL, and pTau181 positivity was defined as CSF pTau181 > 27 pg/mL. * Tau PET data was available for 130 individuals. Abbreviations: Aβ42, amyloid-beta 42; pTau181, phosphorylated tau at threonine 181.

Based on CSF pTau181 levels, 378 individuals were classified as pTau181 negative (pTau181-) and 105 as pTau181 positive (pTau181+). Compared with the pTau181- group, participants in the pTau181+ group were older, more likely to be APOE ε4 carriers, and had smaller baseline total hippocampal volumes (*p* = 0.041), whereas baseline hippocampal asymmetry did not differ between the groups (*p* = 0.245). There was no significant difference in total hippocampal volume or hippocampal asymmetry between the amyloid-positive (Aβ+) and amyloid-negative (Aβ-) groups.

Observed mean longitudinal changes from baseline in total hippocampal volume and hippocampal asymmetry over 10 years of follow-up in the overall cohort are shown in Figure 1. Overall, total hippocampal volume progressively declined over time, whereas hippocampal asymmetry remained relatively stable.

**Fig. 1:**
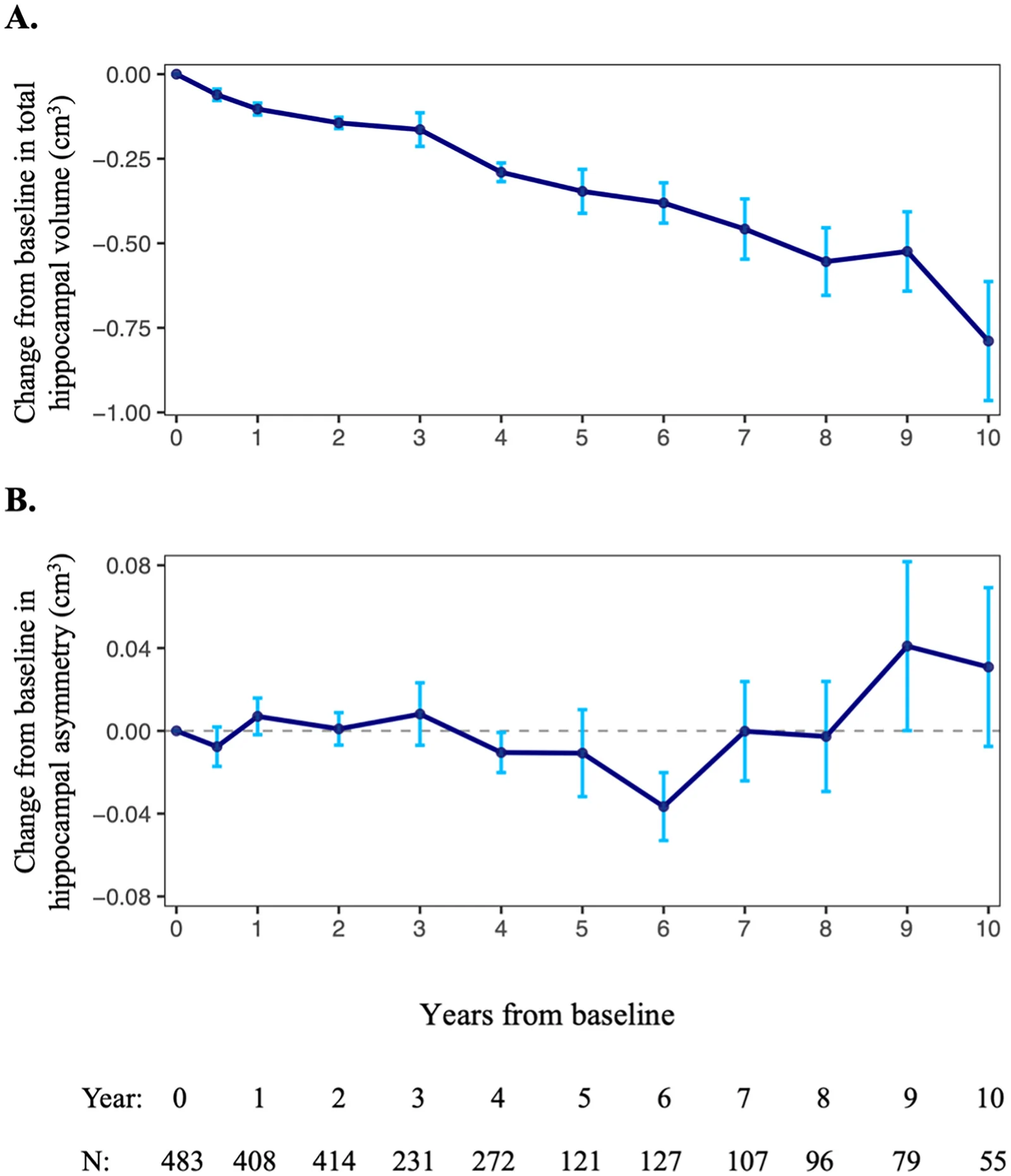
Mean change from baseline in total hippocampal volume (A) and hippocampal asymmetry (B) in the overall cohort across follow-up visits. For each participant, values at each visit were expressed as the difference from their baseline measurement. Points represent the mean across participants at each time point, and error bars indicate the standard error of the mean. Positive values indicate an increase relative to baseline, whereas negative values indicate a decrease.

### 3.2 Association of baseline pTau181 with hippocampal asymmetry over time

In the base model (model 1), higher pTau181 levels were significantly associated with faster increase in hippocampal asymmetry (β = 1.20 ± 0.43, *p* = 0.006, Table 2). These associations remained significant after adjustment for baseline total hippocampal volume (model 2), and further for baseline hippocampal volume, and Aβ42 and its interaction with time (model 3, both *p* = 0.006). Higher pTau181 levels were significantly associated with a faster decline in hippocampal total volume (model 1, β = -1.87 ± 0.60, *p* = 0.002, Table 3). These associations remained significant after adjustment for Aβ42 (model 2), and further for baseline hippocampal asymmetry (model 3). No significant associations were found between pTau181 and hippocampal asymmetry and total volume at baseline.

**Table 2:** Associations of CSF biomarkers with baseline hippocampal asymmetry and longitudinal change.

	pTau181				Aβ42			
	pTau181 β (SE)	p	pTau181 × Time β (SE)	p	Aβ42 β (SE)	p	Aβ42 × Time β (SE)	p
Model 1	0.08 (1.96)	0.967	1.20 (0.43)	**0.006**	-0.01 (0.03)	0.709	-0.00 (0.01)	0.752
Model 2	0.04 (1.96)	0.982	1.20 (0.43)	**0.006**	-0.01 (0.03)	0.715	-0.00 (0.01)	0.751
Model 3	0.22 (1.99)	0.913	1.23 (0.43)	**0.006**	-0.01 (0.03)	0.674	0.00 (0.01)	0.546

Model 1 adjusted for age, sex, education, APOE ε4 status, time, the baseline CSF biomarker (pTau181 or Aβ42), and the corresponding biomarker × time interaction.

Model 2 additionally adjusted for baseline total hippocampal volume.

Model 3 additionally adjusted for the other CSF biomarker (Aβ42 in pTau181 models and pTau181 in Aβ42 models) and its interaction with time.

Abbreviations: Aβ42, amyloid-beta 42; pTau181, phosphorylated tau at threonine 181.

**Table 3:** Associations of CSF biomarkers with baseline total hippocampal volume and longitudinal change.

	pTau181				Aβ42			
	pTau181 β (SE)	P	pTau181 × Time β (SE)	p	Aβ42 β (SE)	p	Aβ42 × Time β (SE)	p
Model 1	2.13 (4.16)	0.609	-1.87 (0.60)	**0.002**	-0.04 (0.06)	0.485	0.03 (0.01)	**0.002**
Model 2	2.54 (4.23)	0.547	-1.96 (0.59)	**0.001**	-0.04 (0.06)	0.448	0.03 (0.01)	**< 0.001**
Model 3	2.50 (4.23)	0.556	-1.96 (0.59)	**0.001**	-0.04 (0.06)	0.451	0.03 (0.01)	**< 0.001**

Model 1 adjusted for age, sex, education, APOE ε4 status, time, the baseline CSF biomarker (pTau181 or Aβ42), and the corresponding biomarker × time interaction.

Model 2 additionally adjusted for the alternate CSF biomarker and its interaction with time.

Model 3 additionally adjusted for baseline hippocampal asymmetry.

Abbreviations: Aβ42, amyloid-beta 42; pTau181, phosphorylated tau at threonine 181.

In contrast, there were no significant associations between baseline Aβ42 levels and hippocampal asymmetry over time (Table 2). However, higher baseline amyloid burden was significantly associated with a faster rate of total hippocampal atrophy over time (β = −0.03± 0.01, *p* = 0.002, Table 3). These associations remained significant after controlling for baseline pTau181. Complete fixed-effects estimates for all linear mixed-effects models in section 3.2, including all covariates, are provided in the Supplementary Tables S1–S9.

### 3.3 Effect of time on hippocampal atrophy and asymmetry

In the same linear mixed-effects models reported in section 3.2., there was a significant effect of time on hippocampal volumes. Total hippocampal volume declined over 10 years of follow-up (β_{time} = −0.07, *p* < 0.001, Table S9). In contrast, hippocampal asymmetry did not show a significant overall change over time (β_{time} = −0.02, *p* = 0.106, Table S5), suggesting that absolute left–right hippocampal volumetric differences remained relatively stable after adjusting for baseline covariates. Model-predicted population-average trajectories of total hippocampal volume and hippocampal asymmetry are presented in Fig. 2A and B, respectively.

**Fig. 2:**
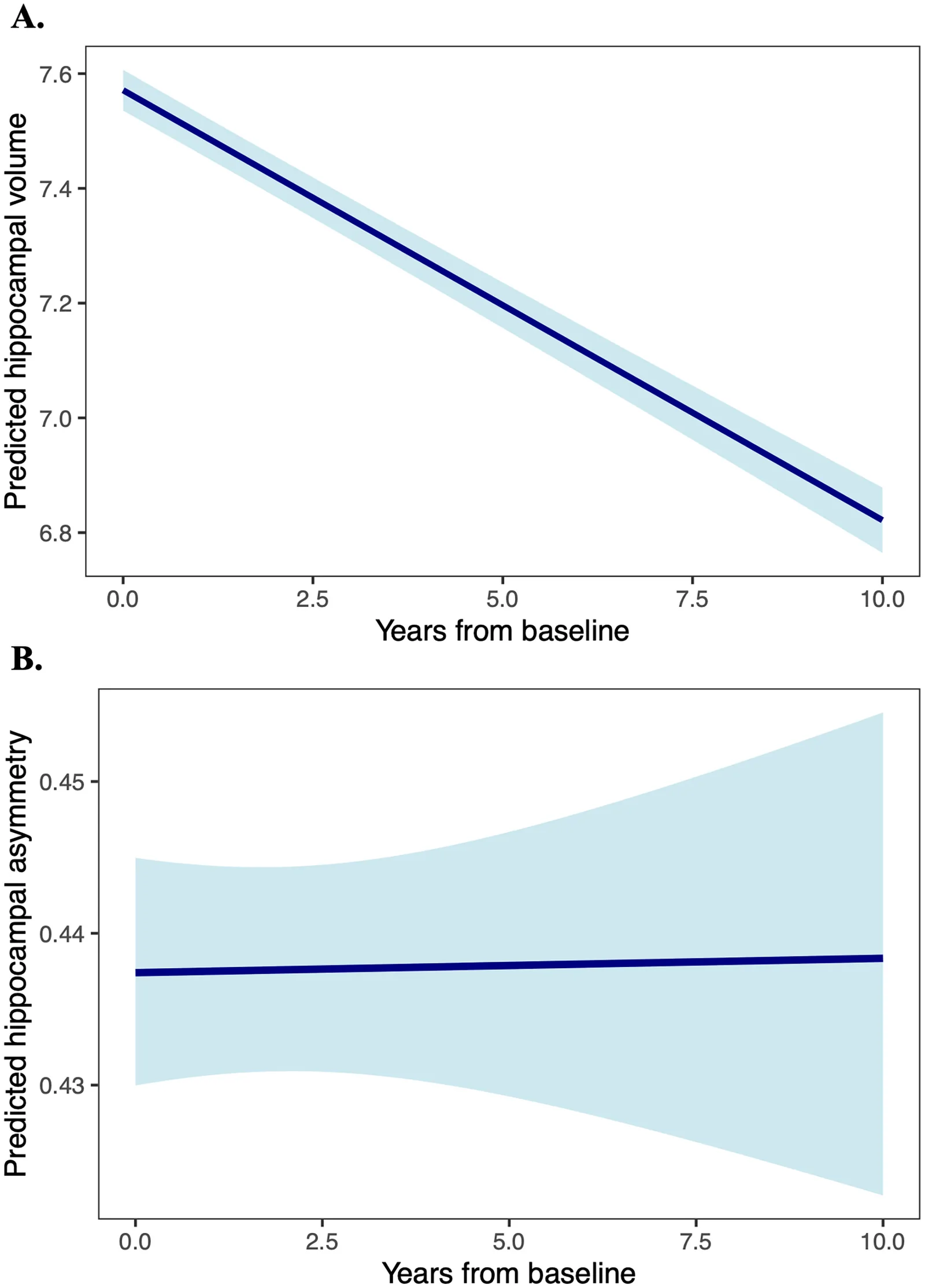
Model-predicted longitudinal trajectories of total hippocampal volume (A) and hippocampal asymmetry (B) over time. Lines represent estimated values from linear mixed-effects models. Shaded areas represent ±1 standard error of the mean. Models were adjusted for age, sex, education, APOE ε4 status, time, CSF biomarkers and their interactions with time; models of hippocampal asymmetry were additionally adjusted for baseline total hippocampal volume.

For visualization purposes only, we fit separate linear mixed-effect models using dichotomized pTau181 and Aβ42 status, while adjusting for the same covariates included in the main models described in section 3.2. The trajectories shown in Figures 3A and 3B represent model-predicted population-average values derived from these models. Although the biomarker × time interaction terms were not statistically significant in these models, possibly due to the loss of information associated with dichotomization, the model-predicted trajectories suggested greater divergence in longitudinal hippocampal asymmetry according to pTau181 status than according to Aβ42 status.

**Fig. 3:**
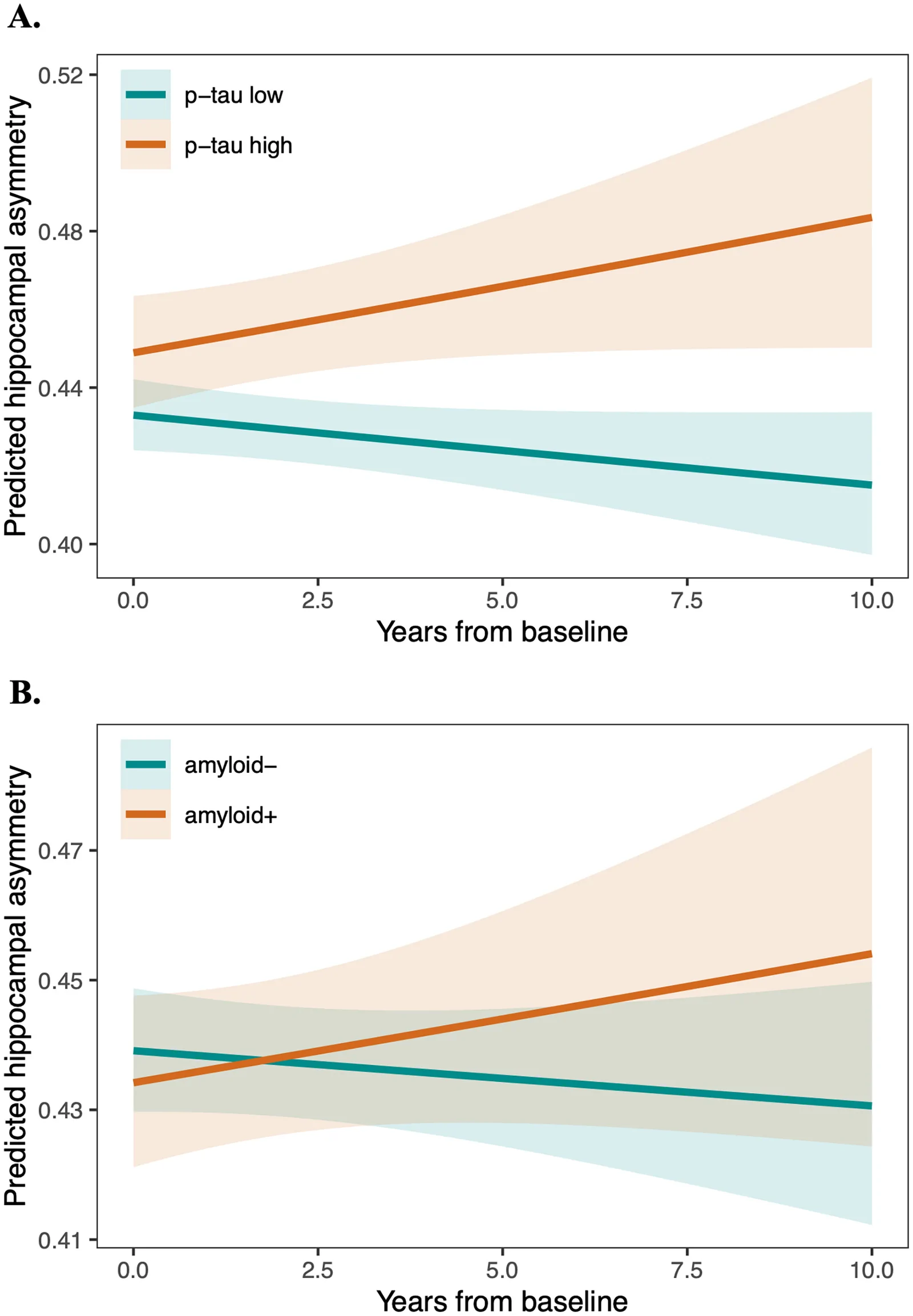
Model-predicted longitudinal trajectories of hippocampal asymmetry by binary baseline pTau181 (A) and Aβ42 (B) status. Lines represent estimated values from linear mixed-effects models for participants with lower (0) and higher (1) levels of each biomarker at baseline. Shaded areas represent ±1 standard error of the mean. Model was adjusted for age, sex, education, APOE ε4 status, time, CSF biomarkers and their interactions with time, and baseline hippocampal volume. Amyloid positivity was defined as CSF Aβ42 < 977 pg/mL, and pTau181 positivity was defined as CSF pTau181 > 27 pg/mL.

In separate models fitted to evaluate the association between pTau181 and longitudinal trajectories of left and right hippocampal volumes, pTau181 was significantly associated with faster longitudinal decline in both hippocampi (β = −0.99, SE = 0.32, *p* = 0.002 for the left, and β = −0.80, SE = 0.33, *p* = 0.015 for the right).

### 3.4 Association of pTau181 with hippocampal asymmetry and total volume stratified by amyloid status

Although the amyloid × time interaction was not statistically significant in the main models reported in section 3.2., to further investigate the effect of amyloid on the association of pTau181 and hippocampal asymmetry, we conducted exploratory analyses by dichotomous Aβ status (Table 4). Higher pTau181 levels were associated with faster increase in hippocampal asymmetry over time only among Aβ- individuals (β = 1.41, SE = 0.55, *p* = 0.014), while in the Aβ+ group no significant associations were observed. In contrast, higher pTau181 levels were associated with faster total hippocampal volume atrophy only among Aβ+ individuals (β = -2.12, SE = 0.61, *p* < 0.001), and not in the Aβ- group. Complete fixed-effects estimates for all linear mixed-effects models in section 3.4, including all covariates, are provided in the Supplementary Tables S10–S13.

**Table 4:** Association baseline CSF pTau181 with hippocampal measures, stratified by baseline amyloid status.

		Hippocampal Asymmetry		Total Hippocampal Volume	
Amyloid status	Term	β (SE)	P value	β (SE)	P value
Aβ -	pTau181	2.71 (2.74)	0.323	1.85 (5.94)	0.755
	pTau181 × time	1.41 (0.55)	**0.014**	-0.71 (0.61)	0.245
Aβ +	pTau181	-1.13 (2.94)	0.701	3.40 (6.04)	0.575
	pTau181 × time	0.11 (0.73)	0.884	-2.12 (0.61)	**< 0.001**

Models included covariates (age, sex, education, APOE4 status), as well as pTau181, time, and pTau181 × time interaction. For analyses of hippocampal asymmetry, baseline hippocampal volume was additionally included as a covariate. Amyloid positivity was defined as CSF Aβ42 < 977 pg/mL.

Abbreviations: Aβ42, amyloid-beta 42; pTau181, phosphorylated tau at threonine 181.

### 3.5 Comparison of pTau181, total tau, and tau PET

In this cohort, CSF pTau181 and total tau (t-tau) were highly correlated (Spearman’s ρ = 0.98, *p* < 0.001). Consistent with this, the association of t-tau with longitudinal changes in hippocampal asymmetry (β = 0.11 ± 0.04, *p* = 0.015) was similar to that observed for pTau181 (β = 1.23 ± 0.43, *p* = 0.006) in the full model including all covariates.

Among 130 of 483 participants with baseline tau PET, temporal meta-ROI SUVR was modestly and significantly correlated with CSF pTau181 (Spearman’s ρ = 0.25, *p* = 0.004) and t-tau (Spearman’s ρ = 0.23, *p* = 0.009). After adjusting for age, sex, and CSF Aβ42, these associations were stronger, with partial correlation coefficient of r = 0.37 for pTau181 and r = 0.33 for t-tau, both *p* < 0.001.

## 4 Discussion

In this longitudinal study of cognitively unimpaired individuals, we found that higher baseline CSF pTau181 was associated with faster increase in hippocampal asymmetry over time, independent of baseline total hippocampal volume and amyloid burden. Higher baseline pTau181 was also associated with increased total hippocampal atrophy, confirming its established relationship with neurodegeneration. In contrast, higher amyloid burden was associated with longitudinal decline in total hippocampal volume but not with change in hippocampal asymmetry. In stratified analyses, the association between pTau181 and increasing asymmetry remained significant in Aβ− individuals, while the association between pTau181 and faster total hippocampal atrophy was significant in Aβ+ individuals. Together, these findings suggest that CSF pTau181 is associated not only with overall hippocampal tissue loss but also with the pattern by which that tissue loss unfolds across hemispheres, and that these two dimensions of neurodegeneration may relate differently to amyloid status.

These findings have potential translational implications. Hippocampal asymmetry may complement conventional measures of total hippocampal atrophy by identifying individuals with distinct patterns of tau-related neurodegeneration at the preclinical stage of AD. Such information may improve biological risk stratification, enhance characterization of disease heterogeneity, and support more biologically informed participant selection for future clinical trials. Furthermore, the integration of CSF biomarkers with repeated MRI measures over extended follow-up enables a more comprehensive characterization of longitudinal neurodegenerative trajectories.

Our findings suggest that hippocampal asymmetry may represent a distinct feature of hippocampal neurodegeneration rather than merely a secondary descriptor of hippocampal atrophy. Total hippocampal volume is a bilateral summary measure of neurodegenerative burden, whereas asymmetry captures left-right imbalance and therefore may index selective or lateralized vulnerability within the MTL. That distinction is important because two individuals may have similar total hippocampal volumes but differ substantially in how degeneration is distributed across hemispheres. Our data suggest that this lateralized component is not simply noise around the global atrophy signal but may represent a biologically meaningful dimension of neurodegeneration. Prior literature on hippocampal asymmetry in AD has been mixed, with some studies reporting limited evidence for disease-related asymmetry and more recent synthesis suggesting greater left-sided volume reduction across AD and MCI groups, underscoring that asymmetry is not a uniform feature of disease severity and may instead reflect subtype- or pathology-specific processes [5]

In a recent work, we have shown that while total hippocampal volume was associated with cognitive performance in multiple domains, greater hippocampal asymmetry was mainly associated with accelerated memory decline, especially among Aβ- individuals [4] That prior observation is important here because it suggests that asymmetry is not merely an alternative structural summary, but a phenotype with distinct cognitive correlates. The current findings extend that work by linking asymmetric degeneration specifically to pTau181.

Previous studies have shown that spatially and temporally, tau pathology correlates much more strongly than amyloid with neurodegeneration and cognitive impairment [22] It has been shown that tau burden had both direct and atrophy-mediated effects on cognition, and these associations were weakly related to amyloid burden [23] Although tau pathology is best assessed using imaging modalities, several studies have demonstrated that CSF pTau181 levels closely reflect cerebral tau burden [[24], [25], [26]]

The link between CSF pTau181 and global hippocampal atrophy has been previously reported [27] In a well-defined AD cohort, CSF total tau and pTau181 were inversely associated with hippocampal volume, whereas CSF Aβ42 was not associated with any brain measure, consistent with neurodegeneration driven by tau pathology in AD [28] In ADNI, CSF pTau181 predicts faster hippocampal atrophy particularly among individuals with mild cognitive impairment and AD,[29] and pTau181 demonstrated the strongest association with hippocampal atrophy, whereas Aβ42 showed the weakest [30] Similarly, an association between CSF pTau181and accelerated hippocampal atrophy has been demonstrated in cognitively normal older adults in ADNI [31] Our findings extend this literature in two ways. First, they confirm in cognitively unimpaired individuals that pTau181 is associated with faster decline in total hippocampal volume even after accounting for amyloid. Second, they suggest that tau-related neurodegeneration may not be fully captured by total hippocampal volume alone, because pTau181 was also associated with increasing hippocampal asymmetry over time.

Importantly, our findings position hippocampal asymmetry as a distinct and potentially informative neuroimaging phenotype. The observation that asymmetry remained relatively stable at the group level but diverged according to baseline pTau181 levels suggests that asymmetric atrophy is not simply a byproduct of global atrophy, but rather reflects an individualized trajectory influenced by underlying pathology.

The differential patterns observed in amyloid-stratified analyses provide further information on the underlying biological mechanisms of p-tau-asymmetry associations. However, these subgroup findings should be interpreted cautiously. Because the statistical significance of an association in one subgroup and not in another does not by itself establish a significant between-group difference, the present results should be viewed as suggestive rather than definitive evidence of effect modification by amyloid status. Even so, the pattern is biologically interesting as pTau181 was associated with increasing hippocampal asymmetry in Aβ- individuals, and with total hippocampal atrophy in Aβ+ individuals. One possible interpretation is that in Aβ+ individuals, pTau181 reflects a more canonical AD-related neurodegenerative process expressed primarily as overall hippocampal tissue loss, whereas in Aβ− individuals pTau181 may be indexing a more regionally selective medial temporal process that manifests as increasing asymmetry. This interpretation is consistent with recent longitudinal works showing the association of tau with hippocampal atrophy, independent of amyloid, in cognitively normal older adults and different downstream structural pathways for tau in Aβ− versus Aβ+ individuals [32,33]

One potential explanation for our findings is the contribution of other age-related co-pathologies that preferentially affect medial temporal structures. Conditions such as primary age-related tauopathy (PART) are common in cognitively unimpaired older adults and is characterized by tau accumulation in the MTL in the absence of significant amyloid pathology [[34], [35], [36]] Similarly, LATE and hippocampal sclerosis of aging often co-occur with tau pathology in older adults and are characterized by MTL-predominant neurodegeneration that may be disproportionate or asymmetric [37,38] Late-life hippocampal sclerosis, in particular, is often asymmetric or unilateral at autopsy, and has been associated with disproportionately severe hippocampal atrophy [39] Although we do not have in-vivo measures to quantity these pathologies, the observed association between pTau181 and asymmetric atrophy in Aβ- individuals is compatible with the possibility that hippocampal asymmetry reflects non-amyloid predominant MTL neurodegeneration, potentially arising from mixed or overlapping age-related pathologies rather than AD neuropathologic change alone.

The relatively modest association between CSF pTau181 and tau PET observed in this study likely reflects both biological and cohort-specific factors. CSF pTau181 is thought to capture early soluble tau dysregulation, whereas tau PET reflects later-stage regional fibrillar tau deposition [40] In addition, the predominance of cognitively unimpaired, CSF pTau181-negative participants likely limited the magnitude and variability of tau PET signal, thereby reducing the strength of their association.

This study has several strengths. We compared total hippocampal volume and asymmetry as two related but potentially distinct structural expressions of neurodegeneration that demonstrate different associations with AD biomarkers. In addition, the focus on cognitively unimpaired individuals enhances the relevance of these findings to the preclinical stages of AD. A few limitations should be considered. First, the absence of in vivo markers of non-AD pathologies, such as TDP-43, precludes direct attribution of asymmetric atrophy to specific underlying mechanisms. Second, this study examined the association between CSF pTau181, a soluble marker of tau pathology, and longitudinal hippocampal asymmetry. Future studies incorporating tau PET, which measures regional aggregated tau, are needed to determine whether regional tau deposition exhibits similar or stronger associations with hippocampal asymmetry, thereby providing additional insight into the mechanisms underlying asymmetric neurodegeneration. Finally, the ADNI cohort is a highly selected population that is predominantly White, highly educated, and has relatively few comorbidities. As a result, our findings may not be fully generalizable to more diverse clinical populations. Given emerging evidence that AD biomarkers and their clinical associations may vary across racial and ethnic groups, future studies in more representative cohorts are needed to evaluate the generalizability of these findings.

In conclusion, baseline CSF pTau181 was associated with both faster total hippocampal atrophy and increasing hippocampal asymmetry over time, whereas amyloid burden was associated with overall hippocampal atrophy but not with asymmetry. These results support the idea that the magnitude and the lateralization of hippocampal neurodegeneration may reflect partially distinct biological processes. Measures of hippocampal asymmetry may therefore add information beyond total hippocampal volume, particularly in cognitively unimpaired older adults in whom non-amyloid-predominant medial temporal degeneration may already be emerging. Incorporating measures of hemispheric asymmetry into neuroimaging frameworks could improve characterization of early and heterogeneous pathways of neurodegeneration in aging and AD.

## Funding resources

This study was supported in part by grants from the National Institute of Health (NIA K23 AG063993; R01AG080635; R01AG095017); the Alzheimer’s Association (SG-24-988292 ISAVRAD); Cure Alzheimer’s Fund.

## Declaration of conflicting interest

All authors declare that they have no commercial or financial relationships that could be construed as a potential conflict of interest. The study received no commercial funding. Dr. Ali Ezzati reports research support from the National Institute on Aging (NIA) and the Alzheimer's Association, as stated in the Fundings. All other authors report no competing interests related to the content of this manuscript.

## Declaration of the use of generative AI

We used AI for writing fluency.

## CRediT authorship contribution statement

**Elham Ghanbarian:** Writing – review & editing, Writing – original draft, Visualization, Methodology, Formal analysis, Data curation, Conceptualization. **Lukai Zheng:** Writing – review & editing. **Tianchen Qian:** Writing – review & editing. **Crystal M. Glover:** Writing – review & editing. **S. Ahmad Sajjadi:** Writing – review & editing. **Maria M. Corrada:** Writing – review & editing. **Joshua D. Grill:** Writing – review & editing. **Ali Ezzati:** Writing – review & editing, Supervision, Investigation, Funding acquisition, Conceptualization.

## Declaration of competing interest

The authors declare the following financial interests/personal relationships which may be considered as potential competing interests:

Ali Ezzati reports financial support was provided by National Institutes of Health, Alzheimer’s Association, Cure Alzheimer’s. If there are other authors, they declare that they have no known competing financial interests or personal relationships that could have appeared to influence the work reported in this paper.
