# Supplementary material for: CSF pTau181 predicts asymmetric hippocampal atrophy in cognitively unimpaired older adults
Source: J Prev Alzheimers Dis. 2026 Nov 4;13(10):100690. doi: 10.1016/j.tjpad.2026.100690 (PMC13756247; doi:10.1016/j.tjpad.2026.100690)
Supplement: Supplementary file 1 [file mmc1.docx]

**Abbreviations (all tables):** Aβ42, amyloid-beta 42; pTau181, phosphorylated tau at threonine 181; SE, standard error.

**Table S1.** Fixed effect estimates for the associations between pTau181 and hippocampal asymmetry (Model 1)

| Variable | Estimate | SE | p-value |
| --- | --- | --- | --- |
| Intercept | -0.863 | 0.214 | <0.001 |
| Sex (Male) | -0.038 | 0.032 | 0.237 |
| APOE ε4 carriers | 0.006 | 0.035 | 0.868 |
| Age (years) | -0.002 | 0.003 | 0.550 |
| Education (years) | 0.010 | 0.006 | 0.101 |
| Time (years) | -0.026 | 0.010 | 0.012 |
| Baseline pTau181 | 0.082 | 1.959 | 0.967 |
| Time × Baseline pTau181 | 1.199 | 0.430 | 0.006 |

Model 1was adjusted for age, sex, education, APOE ε4 status, time, baseline pTau181 and the pTau181× time interaction.

**Table S2.** Fixed effect estimates for the associations between Aβ42 and hippocampal asymmetry (Model 1)

| Variable | Estimate | SE | p-value |
| --- | --- | --- | --- |
| Intercept | -0.879 | 0.219 | <0.001 |
| Sex (Male) | -0.041 | 0.032 | 0.204 |
| APOE ε4 carriers | 0.005 | 0.036 | 0.898 |
| Age (years) | -0.001 | 0.003 | 0.658 |
| Education (years) | 0.010 | 0.006 | 0.098 |
| Time (years) | 0.002 | 0.010 | 0.863 |
| Baseline Aβ42 | -0.010 | 0.027 | 0.709 |
| Time × Baseline Aβ42 | -0.002 | 0.007 | 0.752 |

Model 1 adjusted for age, sex, education, APOE ε4 status, time, baseline Aβ42 and Aβ42 × time interaction.

**Table S3.** Fixed effect estimates for the associations between pTau181 and hippocampal asymmetry (Model 2)

| Variable | Estimate | SE | p-value |
| --- | --- | --- | --- |
| Intercept | -0.998 | 0.323 | 0.002 |
| Sex (Male) | -0.034 | 0.033 | 0.303 |
| APOE ε4 carriers | 0.005 | 0.035 | 0.879 |
| Age (years) | -0.001 | 0.003 | 0.726 |
| Education (years) | 0.011 | 0.006 | 0.091 |
| Time (years) | -0.026 | 0.010 | 0.012 |
| Baseline pTau181 | 0.043 | 1.961 | 0.982 |
| Time × Baseline pTau181 | 1.200 | 0.430 | 0.006 |
| Baseline Hippocampal Volume | 0.011 | 0.020 | 0.579 |

Model 2 was adjusted for age, sex, education, APOE ε4 status, time, baseline pTau181 and the pTau181× time interaction, and baseline hippocampal volume.

**Table S4.** Fixed effect estimates for the associations between Aβ42 and hippocampal asymmetry (Model 2)

| Variable | Estimate | SE | p-value |
| --- | --- | --- | --- |
| Intercept | -1.016 | 0.327 | 0.002 |
| Sex (Male) | -0.037 | 0.033 | 0.266 |
| APOE carrier | 0.004 | 0.036 | 0.908 |
| Age (years) | -0.001 | 0.003 | 0.840 |
| Education (years) | 0.011 | 0.006 | 0.088 |
| Time (years) | 0.002 | 0.010 | 0.864 |
| Baseline Aβ42 | -0.010 | 0.027 | 0.715 |
| Time × Baseline Aβ42 | -0.002 | 0.007 | 0.751 |
| Baseline Hippocampal Volume | 0.011 | 0.020 | 0.572 |

Model 2 was adjusted for age, sex, education, APOE ε4 status, time, baseline Aβ42 and Aβ42 × time interaction, and baseline hippocampal volume.

**Table S5.** Fixed effect estimates for the associations between pTau181 and Aβ42 and hippocampal asymmetry (Model 3)

| Variable | Estimate | SE | p-value |
| --- | --- | --- | --- |
| Intercept | −0.964 | 0.328 | 0.003 |
| APOE ε4 non-carriers | 0.014 | 0.037 | 0.699 |
| Sex (Male) | −0.034 | 0.033 | 0.303 |
| Age (years) | −0.001 | 0.003 | 0.634 |
| Education (years) | 0.011 | 0.006 | 0.088 |
| Time (years) | −0.021 | 0.013 | 0.106 |
| Baseline pTau181 | 0.216 | 1.989 | 0.913 |
| Time × Baseline pTau181 | 1.232 | 0.434 | 0.006 |
| Baseline Aβ42 | −0.011 | 0.027 | 0.674 |
| Time × Baseline Aβ42 | −0.004 | 0.006 | 0.546 |
| Baseline hippocampal volume | 0.010 | 0.020 | 0.589 |

Model 3 was adjusted for age, sex, education, APOE ε4 status, time, baseline CSF biomarkers (pTau181 and Aβ42) and their interaction with time, and baseline hippocampal volume.

| Variable | Estimate | SE | p-value |
| --- | --- | --- | --- |
| Intercept | 12.388 | 0.493 | <0.001 |
| APOE ε4 carrier | 0.078 | 0.081 | 0.338 |
| Sex (Male) | −0.360 | 0.072 | <0.001 |
| Age (years) | −0.057 | 0.006 | <0.001 |
| Education (years) | −0.030 | 0.014 | 0.031 |
| Time (years) | −0.110 | 0.012 | <0.001 |
| Baseline Aβ42 | −0.039 | 0.056 | 0.485 |
| Time × Baseline Aβ42 | 0.026 | 0.008 | 0.002 |

**Table S6.** Fixed effect estimates for the associations between Aβ42 and total hippocampal volume (Model 1)

Model 1 was adjusted for age, sex, education, APOE ε4 status, time, baseline Aβ42, and Aβ42 × time interaction.

**Table S7.** Fixed effect estimates for the associations between pTau181 and total hippocampal volume (Model 1)

| Variable | Estimate | SE | p-value |
| --- | --- | --- | --- |
| Intercept | 12.303 | 0.481 | <0.001 |
| Sex (Male) | -0.358 | 0.073 | <0.001 |
| APOE ε4 carriers | 0.070 | 0.078 | 0.368 |
| Age (years) | -0.057 | 0.006 | <0.001 |
| Education (years) | -0.031 | 0.014 | 0.029 |
| Time (years) | -0.035 | 0.014 | 0.013 |
| Baseline pTau181 | 2.130 | 4.156 | 0.609 |
| **Time × Baseline pTau181** | **-1.871** | **0.600** | **0.002** |

Model 1 was adjusted for age, sex, education, APOE ε4 status, time, baseline pTau181, and pTau181 × time interaction.

**Table S8.** Fixed effect estimates for the associations between Aβ42 and pTau181and total hippocampal volume (Model 2)

| Variable | Estimate | SE | p-value |
| --- | --- | --- | --- |
| Intercept | 12.378 | 0.495 | <0.001 |
| APOE ε4 carrier | 0.084 | 0.073 | 0.316 |
| Sex (Male) | −0.356 | 0.084 | <0.001 |
| Age (years) | −0.057 | 0.006 | <0.001 |
| Education (years) | −0.031 | 0.014 | 0.031 |
| Time (years) | −0.070 | 0.017 | <0.001 |
| Baseline Aβ42 | −0.043 | 0.057 | 0.448 |
| Time × Baseline Aβ42 | 0.027 | 0.008 | <0.001 |
| Baseline pTau181 | 2.544 | 4.226 | 0.547 |
| Time × Baseline pTau181 | −1.965 | 0.593 | 0.001 |

Model 2 was adjusted for age, sex, education, APOE ε4 status, time, baseline CSF biomarkers (pTau181 and Aβ42) and their interaction with time.

**Table S9.** Fixed effect estimates for the associations between Aβ42 and pTau181and total hippocampal volume (Model 3)

| Variable | Estimate | SE | p-value |
| --- | --- | --- | --- |
| Intercept | 12.371 | 0.496 | <0.001 |
| APOE ε4 non-carriers | 0.084 | 0.084 | 0.321 |
| Sex (Male) | −0.357 | 0.073 | <0.001 |
| Age (years) | −0.057 | 0.006 | <0.001 |
| Education (years) | −0.031 | 0.014 | 0.030 |
| Time (years) | −0.070 | 0.017 | <0.001 |
| Baseline Aβ42 | -0.043 | 0.057 | 0.451 |
| Time × Baseline Aβ42 | 0.027 | 0.008 | <0.001 |
| Baseline pTau181 | 2.496 | 4.234 | 0.556 |
| Time × Baseline pTau181 | −1.964 | 0.593 | 0.001 |
| Baseline hippocampal asymmetry | 0.053 | 0.209 | 0.799 |

Model 3 was adjusted for age, sex, education, APOE ε4 status, time, baseline CSF biomarkers (pTau181 and Aβ42) and their interaction with time, and baseline hippocampal asymmetry.

**Table S10.** Fixed effect estimates for the associations between pTau181and hippocampal asymmetry in amyloid-negative participants

| Variable | Estimate | SE | p-value |
| --- | --- | --- | --- |
| Intercept | -1.348 | 0.39 | <0.001 |
| APOE ε4 non-carriers | -0.001 | 0.05 | 0.973 |
| Sex (Male) | -0.058 | 0.039 | 0.141 |
| Age (years) | -0.000 | 0.003 | 0.779 |
| Education (years) | 0.015 | 0.007 | 0.037 |
| Time (years) | -0.032 | 0.012 | 0.013 |
| Baseline pTau181 | 2.708 | 2.737 | 0.323 |
| Time × Baseline pTau181 | 1.411 | 0.554 | **0.014** |
| Baseline hippocampal volume | 0.041 | 0.024 | 0.092 |

**Table S11.** Fixed effect estimates for the associations between pTau181and hippocampal asymmetry in amyloid-positive participants

| Variable | Estimate | SE | p-value |
| --- | --- | --- | --- |
| Intercept | -0.489 | 0.572 | 0.394 |
| APOE ε4 non-carriers | -0.013 | 0.058 | 0.828 |
| Sex (Male) | 0.014 | 0.059 | 0.811 |
| Age (years) | -0.001 | 0.005 | 0.810 |
| Education (years) | -0.002 | 0.005 | 0.826 |
| Time (years) | 0.006 | 0.018 | 0.719 |
| Baseline pTau181 | -1.133 | 2.944 | 0.701 |
| Time × Baseline pTau181 | 0.107 | 0.734 | 0.884 |
| Baseline hippocampal volume | -0.025 | 0.036 | 0.491 |

**Table S12.** Fixed effect estimates for the associations between pTau181and total hippocampal volume in amyloid-negative participants

| Variable | Estimate | SE | p-value |
| --- | --- | --- | --- |
| Intercept | 12.303 | 0.603 | <0.001 |
| APOE ε4 non-carriers | 0.058 | 0.112 | 0.601 |
| Sex (Male) | -0.295 | 0.089 | 0.001 |
| Age (years) | -0.052 | 0.007 | <0.001 |
| Education (years) | -0.049 | 0.017 | 0.004 |
| Time (years) | -0.045 | 0.013 | <0.001 |
| Baseline pTau181 | 1.854 | 5.944 | 0.755 |
| Time × Baseline pTau181 | -0.714 | 0.613 | 0.245 |

**Table S13.** Fixed effect estimates for the associations between pTau181and total hippocampal volume in amyloid-positive participants

| Variable | Estimate | SE | p-value |
| --- | --- | --- | --- |
| Intercept | 12.631 | 0.798 | <0.001 |
| APOE ε4 non-carriers | 0.131 | 0.128 | 0.305 |
| Sex (Male) | -0.430 | 0.124 | <0.001 |
| Age (years) | -0.069 | 0.010 | <0.001 |
| Education (years) | 0.007 | 0.024 | 0.762 |
| Time (years) | -0.039 | 0.015 | 0.011 |
| Baseline pTau181 | 3.395 | 6.038 | 0.575 |
| Time × Baseline pTau181 | -2.121 | 0.606 | **<0.001** |
